# Exploring association between ambient air pollution and glaucoma in China: a nationwide analysis with predictive modeling based on the China Health and Retirement Longitudinal Study

**DOI:** 10.3389/fpubh.2025.1541803

**Published:** 2025-04-25

**Authors:** Xiang Li, Zhan-Yang Luo, Sen Lei, Zhi-Jie Zhang, Jia-feng Tang, Yi-qing Sun

**Affiliations:** ^1^Eye Institute and Affiliated Xiamen Eye Center, School of Medicine, Xiamen University, Xiamen, Fujian, China; ^2^Chongqing Key Laboratory of Development and Utilization of Genuine Medicinal Materials in Three Gorges Reservoir Area, Chongqing Three Gorges Medical College, Wanzhou, China; ^3^Department of Pharmacy, Shanghai Pudong Hospital, Fudan University Pudong Medical Center, Shanghai, China; ^4^Department of Pancreatobiliary Surgery, State Key Laboratory of Oncology in South China, Guangdong Key Laboratory of Nasopharyngeal Carcinoma Diagnosis and Therapy, Guangdong Provincial Clinical Research Center for Cancer, Sun Yat-sen University Cancer Center, Guangzhou, China; ^5^Department of Orthopedics, The First Affiliated Hospital of Xi’an Jiaotong University, Xi'an, Shaanxi, China

**Keywords:** glaucoma, air pollutant, CHARLS, machine learning, middle-aged and older adults

## Abstract

**Background:**

Glaucoma, a leading global cause of blindness, has garnered increasing research attention in recent years concerning its potential association with environmental factors. This study investigates the influence of various environmental pollutants on glaucoma prevalence among middle-aged and older adult populations in China, a country with a high incidence of the disease.

**Methods:**

Using data from 17,184 participants in the 2015 China Health and Retirement Longitudinal Study (CHARLS), individuals were grouped based on glaucoma diagnosis. Environmental pollutant exposure levels were derived from satellite-based spatiotemporal models. Standardized linear regression and restricted cubic spline (RCS) analysis were applied to evaluate the impact of pollutants on glaucoma across different covariate-adjusted models, while logistic regression was used to assess significant variables for constructing and evaluating a predictive model.

**Results:**

After adjusting for covariates, six pollutants (PM_2.5_, PM_10_, PM_1_, NH_4_, Cl, and NO_3_) demonstrated significant associations with glaucoma incidence. Subsequent logistic regression revealed that the occurrence of glaucoma may be influenced by a combination of environmental pollutants (NH_4_ and NO_3_), regional categories, gender, age, educational level, and diabetes history.

**Conclusion:**

In conclusion, this study offers a novel perspective on glaucoma risk prediction by integrating environmental pollutants, potentially contributing to enhanced preventive strategies for this condition.

## Introduction

Glaucoma is a leading cause of irreversible blindness worldwide, characterized by degenerative changes in the optic nerve head and progressive loss of visual field (1). According to the Global Burden of Disease study, glaucoma has been identified as the primary blinding eye disease globally, placing an increasing health burden on human populations. The rising Disability-Adjusted Life Years (DALYs) further underscores this issue. Public data reveal that in China, the prevalence of glaucoma reaches 2.58%, with approximately 21.8 million patients in 2020, accounting for nearly a quarter of the global glaucoma population, and blindness affecting up to 5.67 million individuals (2). Although glaucoma primarily affects the older adult, it is gradually manifesting in younger age groups, posing a significant public health challenge in China with far-reaching social and economic implications. Identifying potential risk factors for glaucoma is therefore essential for early prevention, intervention, and control (3, 4).

Due to its constant exposure to the external environment, the eye is susceptible to external factors such as air pollutants. In recent years, with the worsening of air pollution, researchers have increasingly focused on its potential impact on glaucoma incidence (5, 6). Studies have found that glaucoma incidence is markedly higher in urban than in rural areas, suggesting that air pollution may be a contributing factor (7). Two hospital-based studies indicate that short-term increases in air pollutants (such as PM_2.5_, PM_10_, nitrogen dioxide and carbon monoxide) are significantly associated with increased outpatient visits for acute glaucoma. However, there is a lack of systematic research on the effects of long-term exposure to various air pollutants on glaucoma incidence (8, 9). Existing evidence predominantly stems from isolated urban settings or short-term exposure analyses, often focusing on single pollutants. Such fragmented approaches limit the generalizability of findings and obscure the cumulative effects of complex air pollution mixtures over extended periods.

The China Health and Retirement Longitudinal Study (CHARLS) is a nationally representative dynamic cohort study, recruiting community-dwelling adults aged 45 and older in China (10). In this nationwide cross-sectional study, we aim to utilize CHARLS data to systematically investigate association between long-term exposure to air pollution and glaucoma incidence. Unlike previous fragmented approaches, our study innovatively integrates multi-year environmental exposure data (including PM_2.5_, PM_10_, NO_2_, and CO) with nationally representative health metrics, enabling simultaneous assessment of multiple pollutants’ cumulative effects across diverse geographic regions. By integrating extensive environmental pollution data with population health data, this study provides new evidence to elucidate the potential link between air pollution and glaucoma. Compared to previous studies limited to a single city or short-term exposure, our research offers broader temporal and spatial coverage, lending greater representativeness and validity. Additionally, we aim to develop a predictive model to identify individuals at higher risk of glaucoma, incorporating a range of factors, including air pollution exposure. By integrating these variables into a predictive framework, this model seeks to enhance early detection and risk stratification, providing a practical tool to guide targeted prevention and intervention efforts for high-risk populations. This pioneering study is expected to generate crucial scientific evidence for glaucoma prevention and control in China, while offering valuable insights for environmental protection policy development.

## Methods

### Study design

We utilized publicly available data from CHARLS^1^. Given its large sample size and high quality, the CHARLS data provides robust support for the analyses in this study. CHARLS 2015 data was selected, which originally included 21,038 participants. After excluding individuals with missing data on glaucoma diagnosis, air pollutant exposures, or essential covariates (including age, gender, diabetes, and hypertension), 17,184 participants with complete and analyzable data were retained for the final analysis.

### Assessment of air pollution exposure

Full-coverage ground-level air pollution concentrations (PM_2.5_, PM_10_, PM_1_, O_3_, Cl, NH_4_, NO_3_ and SO_4_) for each individual was assessed by artificial intelligence at 0.1° (≈10 km) gridded spatial resolution from 2015, which were collected from the China High Air Pollutants (CHAP) dataset^2^. Briefly, ground-based measurements, remote sensing products, atmospheric reanalysis, and model simulations were all employed and the space–time extremely randomized trees (STET) model was used to estimate the daily concentrations of ambient PM_2.5_, PM_10_, PM_1_, O_3_, Cl, NH_4_, NO_3_ and SO_4._ Annual air pollution exposure of each participant was estimated based on their county-level residential address. We calculated the mean value, standard deviation, minimum value and maximum value of each air pollutant in 28 provinces included in CHARLS (Supplementary Table S1), and averaged the above data in Table 1. Due to missing data for NH4, SO4, NO3, and Cl in Qinghai and Xinjiang provinces (less than 10%), we applied multiple imputation using the mice package in R to address these gaps. This allowed us to fill in the missing values based on observed data, ensuring a more complete and consistent exposure assessment for these regions in the follow-up analysis.

**Table 1:** Annual average concentrations of air pollutants in 2015.

Air pollutant	Mean	SD	Min	Max
PM_2.5_ (μg/m^3^)	45.10	7.44	27.14	72.40
PM_10_ (μg/m^3^)	81.66	14.90	49.22	131.67
PM_1_ (μg/m^3^)	24.46	4.26	13.95	40.20
O_3_ (μg/m^3^)	82.67	5.80	50.90	109.94
Cl (μg/m^3^)	1.83	0.33	0.93	4.17
NH_4_ (μg/m^3^)	6.08	0.92	3.26	9.30
NO_3_ (μg/m^3^)	8.00	1.52	4.02	13.60
SO_4_ (μg/m^3^)	9.83	1.30	5.75	14.12

### Covariates

The study included numerous covariates covering demographic, socioeconomic, and chronic disease-related factors. Demographic variables comprised age (in years) and sex (“male” or “female”). Socioeconomic variables included residence (“rural” or “urban”), education level (“elementary school or below,” “secondary school,” “high school,” or “university/college”), marital status (“married” or “unmarried”), and region (“east,” “midland,” or “west”). Health behaviors and lifestyles included smoking status (“non-smoker” or “smoker”) and drinking status (“non-drinker” or “drinker”). Chronic disease-related variables covered hypertension, diabetes, and hyperlipidemia, each recorded as “yes” or “no.”

### Diagnostic criteria for glaucoma, hypertension and diabetes

Glaucoma was assessed through self-reports. The presence of glaucoma was defined by the response to the question, “Has a doctor, nurse, or paramedic ever treated you for glaucoma?” If the participant answered “yes, “they were classified as having glaucoma (3). Diabetes was defined based on self-reported physician diagnosis, use of hypoglycemic drugs, fasting blood glucose ≥ 126 mg/dL, and/or glycated hemoglobin ≥ 6.5% at baseline, in line with established diagnostic criteria (11). Hypertension was classified according to the following criteria: (1) self-report of a physician diagnosis in response to the question, “Have you ever been diagnosed with hypertension?”; (2) self-reported use of antihypertensive medications, as indicated by the response to the question, “Are you currently using any antihypertensive medications to manage your blood pressure?”; or (3) confirmation from two or more readings of systolic blood pressure (SBP) ≥ 140 mmHg and/or diastolic blood pressure (DBP) ≥ 90 mmHg (12).

### Statistical analysis of glaucoma and air pollutants

Descriptive statistics were conducted, with continuous variables expressed as mean ± standard deviation (SD) and categorical variables as counts (percentages). Differences in continuous and categorical variables between non-glaucoma and glaucoma participants were analyzed using the *t*-test. Generalized linear models (GLM) were applied to examine relationship between air pollution and glaucoma. We reported effect estimates and 95% confidence intervals (95%CI) as odds ratios (OR). The initial model (Model 1) was unadjusted, while Model 2 adjusted for demographic (age and gender) and socioeconomic variables (regional categories, education, marital status, and drinking status). These factors were selected based on their potential influence on both the exposure to air pollution and the risk of glaucoma, as well as their role as known confounders in health research. Finally, Model 3 further adjusts for the presence of diabetes and hypertension, which are both chronic diseases with well-established links to an increased risk of glaucoma. Given the chronic nature of these conditions, we considered them important confounders, as they may interact with air pollution exposure to influence the development of glaucoma. This model thus provides a more comprehensive adjustment for factors that could affect the observed association between air pollution and glaucoma. Additionally, standardized concentrations of air pollutants were used to compare OR values, identifying the air pollution components most strongly associated with glaucoma. All statistical analyses were conducted using R software (Version 4.4.1). A two-tailed *p*-value < 0.05 was considered statistically significant. To explore the relationship between environmental pollutants and glaucoma, restricted cubic splines (RCS) were employed following standardized linear regression analysis. RCS allows for non-linear relationships while maintaining local structure, revealing pollutant concentration effects on glaucoma risk.

### Evaluation of logistic regression analysis for predictive model construction

Due to the relatively small number of glaucoma cases in our study, we used a *p*-value < 0.10 as a screening threshold in the univariate analysis to avoid prematurely excluding potentially meaningful variables. This more inclusive approach is commonly used in the early stages of model development. For the final multivariate logistic regression model, a *p*-value < 0.05 was used to determine statistical significance. The cohort was divided into training (70%) and validation (30%) datasets. Logistic regression was performed using R (Version 4.4.1).

### Development and validation of predictive model

Given the imbalanced nature of the dataset—with only 1.4% of participants diagnosed with glaucoma—accuracy was not used as a primary evaluation metric due to its limited interpretability in such settings. Instead, we assessed model performance using more robust metrics such as AUC, sensitivity, specificity, and the Brier score. These measures are more appropriate in reflecting the discriminatory power of the model under class imbalance. These metrics provide insights into the ability of model to correctly identify both glaucoma cases and non-cases. To ensure model robustness and avoid overfitting, we employed bootstrapping, where the original dataset was repeatedly sampled 1,000 times for model calibration. Model calibration was further evaluated using the Brier score, calibration curve, and the Hosmer-Lemeshow test, which assess the accuracy of predicted probabilities versus actual outcomes.

Finally, we employed Nomogram and Decision Curve Analysis (DCA) to visualize the prediction of model and assess its clinical utility. The nomogram provides a visual representation of how each variable contributes to the predicted probability of glaucoma, while DCA evaluates the model’s performance in decision-making by considering the net benefit of applying the model across different threshold probabilities. Specifically, for the construction of the nomogram, each variable (including age, gender, education level, diabetes, regional category, NH4 and NO3) was converted into a point value on a standardized “Points” axis. These values were derived from the regression coefficients of the final multivariate logistic model. The total score was then mapped onto a probability scale to estimate the risk of glaucoma. This approach allows variables with different units and scales to be incorporated into a unified predictive model.

## Results

### Descriptive statistics

A total of 21,038 middle-aged and older adults from 28 provinces were initially included, with 17,184 remaining after exclusions. The mean age was 61.48 ± 9.97 years, and 247 participants (1.4%) were diagnosed with glaucoma. Significant differences (*p* < 0.05) between glaucoma and non-glaucoma groups were found in age, marital status, education, drinking status, regional categories, hypertension, and diabetes (Table 2).

**Table 2:** Basic characteristics of participants during the 2015 survey in CHARLS.

Characteristics	Total (*n* = 17,184)	Non-glaucoma (*n* = 16,877)	Glaucoma (*n* = 247)	*p*-value
Age	61.48 ± 9.97	61.39 ± 9.93	67.37 ± 10.70	<0.001
BMI, kg/m^2^	23.87 ± 3.71	23.87 ± 3.70	23.82 ± 3.93	0.770
Gender				
Male	8,130 (47.3)	8,040 (52.2)	90 (36.4)	<0.001
Female	9,004 (52.3)	8,847 (47.4)	157 (63.5)	
Residence				
Rural	10,664 (61.9)	10,486 (61.9)	158 (63.9)	0.543
Urban	6,490 (37.7)	6,401 (37.7)	89 (36.0)	
Marital status				
Married	14,660 (85.3)	14,470 (85.4)	190 (76.9)	0.001
Unmarried	2,468 (14.3)	2,411 (14.2)	57 (23.0)	
Education status				
Elementary school or below	7,659 (44.5)	7,517 (44.3)	142 (57.4)	<0.001
Secondary school	3,791 (22.0)	3,736 (22.0)	55 (22.2)	
High school	3,652 (21.2)	3,618 (21.3)	34 (13.7)	
University/College	2029 (11.8)	2013 (11.8)	16 (6.4)	
Smoking status				
Non-smoker	9,540 (55.5)	9,390 (55.4)	150 (60.7)	0.107
Smoker	7,582 (44.1)	7,485 (44.1)	97 (39.3)	
Drinking status				
Non-drinker	9,261 (53.8)	9,108 (53.7)	153 (61.9)	0.010
Drinker	7,832 (45.5)	7,738 (45.6)	94 (38.1)	
Regional categories				
East	5,968 (34.7)	5,910 (34.8)	58 (23.4)	0.036
Midland	5,566 (32.3)	5,454 (32.2)	112 (45.3)	
West	5,600 (32.5)	5,523 (32.6)	77 (31.1)	
Diabetes				
Yes	1,604 (9.3)	1,568 (9.2)	36 (14.5)	0.019
No	14,640 (85.1)	14,442 (85.2)	198 (80.1)	
Hypertension				
Yes	5,557 (32.3)	5,457 (32.2)	100 (40.4)	0.010
No	10,784 (62.7)	10,648 (68.8)	136 (55.0)	
Hyperlipidemia				
Yes	734 (4.2)	726 (4.2)	8 (3.2)	0.398
No	10,480 (60.9)	10,330 (60.9)	150 (60.7)	

Table 1 present the average concentrations of eight air pollutants. A average ambient PM_2.5_, PM_10_, PM_1_, O_3_, Cl, NH_4_, NO_3_ and SO_4_ exposure were 45.10 ± 7.44 μg/m^3^, 81.66 ± 14.90 μg/m^3^, 24.46 ± 4.26 μg/m^3^, 82.67 ± 5.80 μg/m^3^, 1.83 ± 0.33 μg/m^3^, 6.08 ± 0.92 μg/m^3^, 8.00 ± 1.52 μg/m^3^ and 9.83 ± 1.30 mg/m^3^, respectively. We found that the annual PM_2.5_ and PM_10_ exposure were far greater than the standards of the World Health Organization (AQG 2015: PM_2.5_: 10 μg/m^3^, PM_10_:20 μg/m^3^) and the secondary standard of Chinese ambient air quality guideline (GB 3095–2012, PM_2.5_: 35 μg/m^3^, PM_10_:70 μg/m^3^).

### Association between air pollution and the prevalence of glaucoma

**Figure 1:**
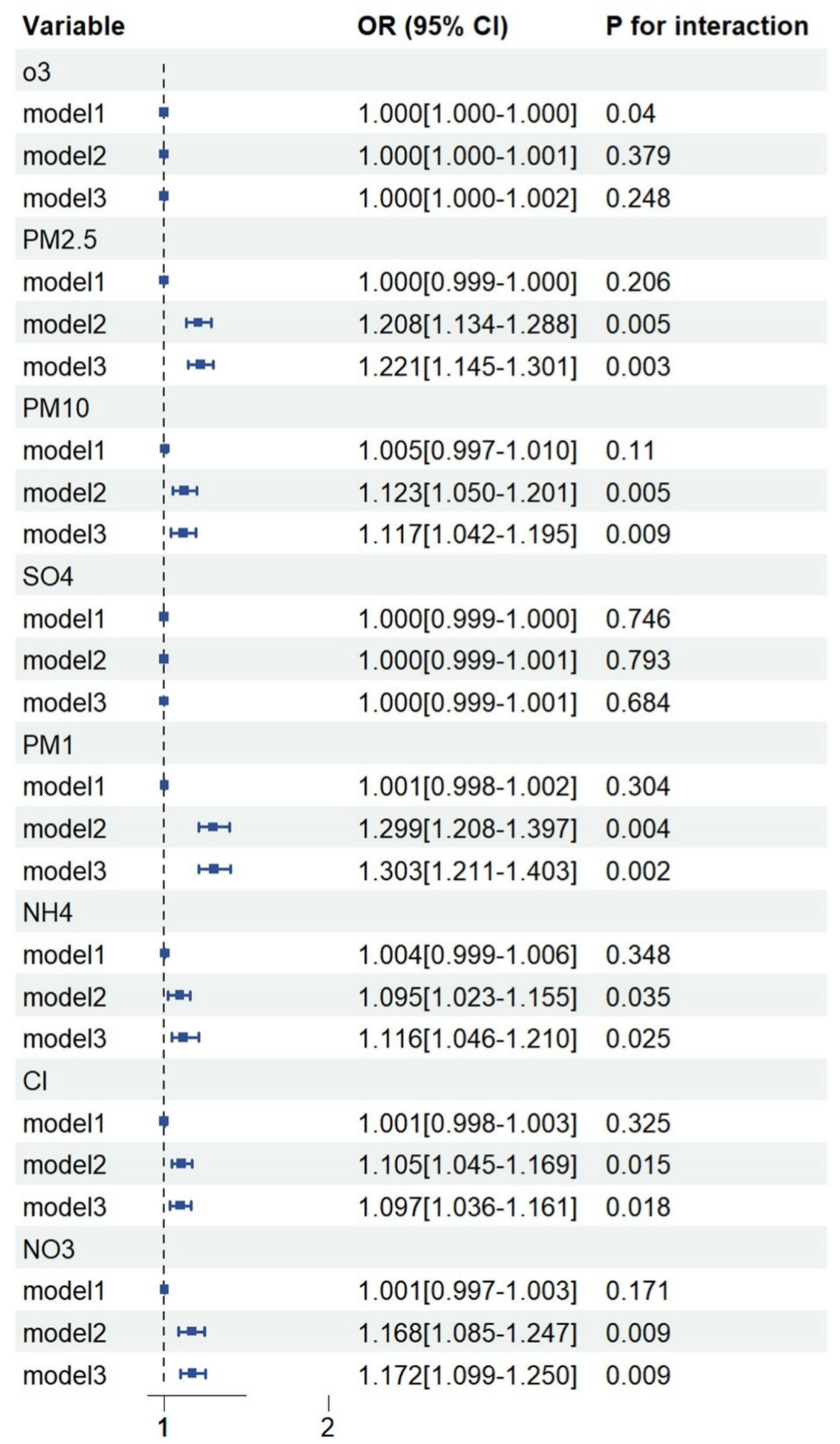
Generalized linear models on exploring associations between air pollution and glaucoma. Model 1, crude model, without adjustment; Model 2, adjusted for gender, age, marital status, drinking status, education status and regional categories; Model 3, adjusted for gender, age, marital status, drinking status, education status and regional categories, diabetes and hypertension.

The associations between each air pollutant and the prevalence of glaucoma are shown in Figure 1. Only O_3_ was associated with increased prevalence of glaucoma in the crude model (model 1). After adjusting for gender, age, marital status, drinking status, education status and regional categories, long-term exposures to ambient PM_1_, PM_2.5_, PM_10_, NH_4_, Cl and NO_3_ were all associated with the increased prevalence of glaucoma in the model 2. Moreover, in the basis of model 2, chronic disease including hypertension and diabetes was added (model 3). Our results found that PM_1_, PM_2.5_, PM_10_, NH_4_, Cl and NO_3_ also associated with glaucoma. Detailed results are shown in Supplementary Table S2. Thus, following analysis based on the model 3 and above six air pollutants.

### Restricted cubic splines

**Figure 2:**
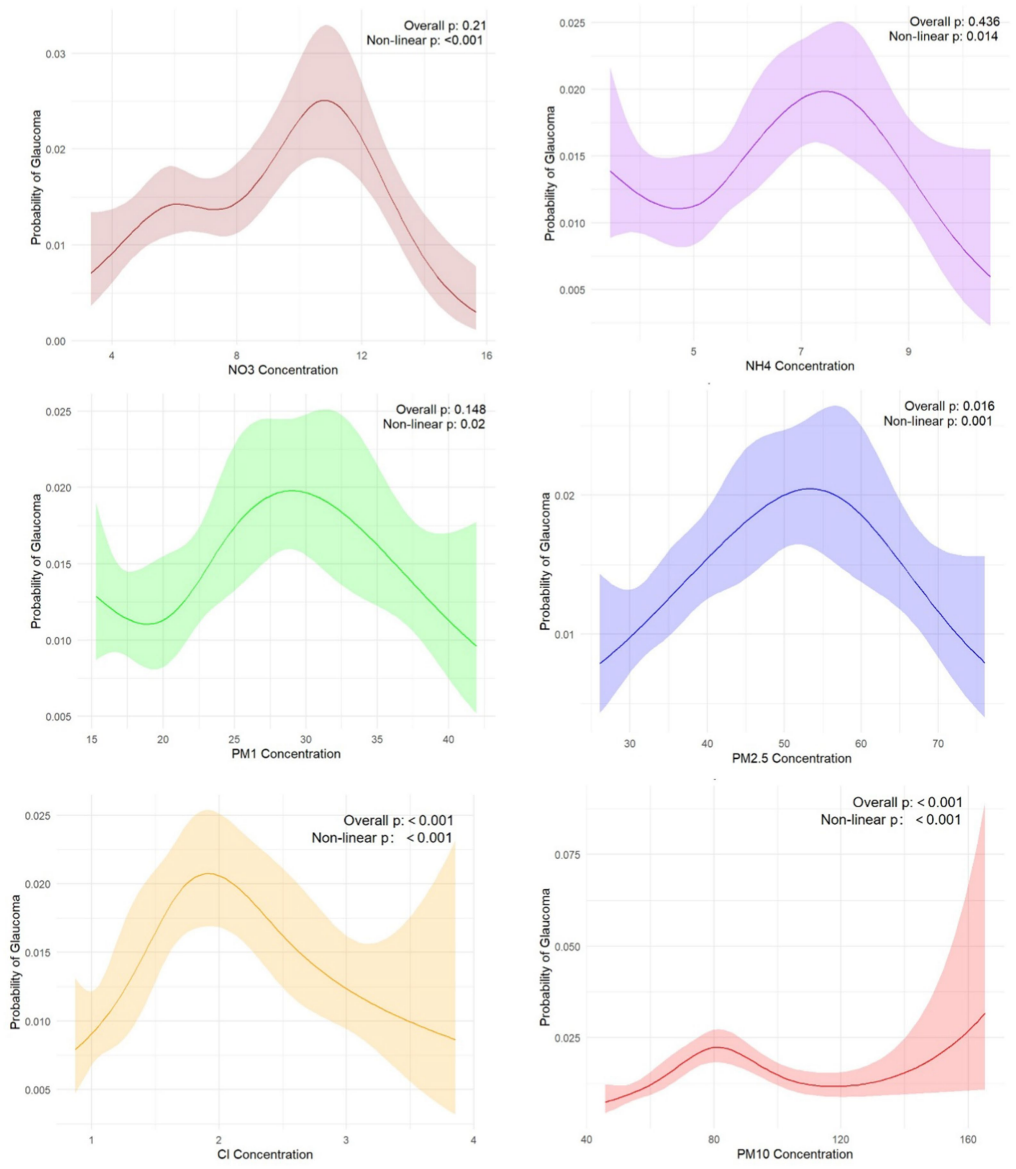
Restricted cubic splines of air pollutants and glaucoma associations.

PM_1_ (for non-linearity, *p* = 0.02)_,_ PM_2.5_ (for non-linearity, *p* < 0.001)_._, PM_10_ (for non-linearity, p < 0.001), NH_4_ (for non-linearity, *p* = 0.014), Cl (for non-linearity, *p* < 0.001) and NO_3_ (for non-linearity, *p* < 0.001) all had an effect on the risk of glaucoma, and the relationship was non-linear. The concentration of different pollutants has different effects on the risk of glaucoma, but overall, the risk of glaucoma increases within a certain range as the concentration increases. However, since most confidence intervals are wide, this may be due to small number of positive samples for glaucoma, increasing the uncertainty in prediction of RCS (Figure 2).

### Construction of predictive model

After determining the factors ultimately included in the model, we conducted logistic regression to explore whether there was a significant relationship between the factors. The specific information of participants in predictive model was listed in Table 3 with 12,108 participants in the training cohort and 5,156 participants in the validation cohort. The results by univariate and multivariate logistic regression analysis in Table 4 showed that gender, age, diabetes, regional categories, education, NH_4_ and NO_3_ were predictors (*p* < 0.1).

**Table 3:** Baseline of validation and training cohort.

Variables	Level	Training cohort (*n* = 12,028)	Validation cohort (*n* = 5,156)	*χ*^2^/*t*-value	*p*-value
PM_1_ [Mean (SD)]		25.59 (7.91)	25.67 (7.84)	1.028	0.304
PM_2.5_ [Mean (SD)]		46.11 (14.14)	46.29 (14.02)	1.265	0.206
PM_10_ [Mean (SD)]		80.13 (25.23)	80.36 (25.02)	1.599	0.110
NH_4_ [Mean (SD)]		6.35 (1.82)	6.37 (1.81)	0.939	0.348
NO_3_ [Mean (SD)]		8.36 (3.25)	8.41 (3.23)	1.370	0.171
Cl [Mean (SD)]		1.78 (0.07)	1.80 (0.06)	0.983	0.325
Age [Mean (SD)]		61.54 (9.94)	61.35 (10.04)	9.379	<0.001
Gender (n)	Female	6,253	2,751	11.744	0.001
	Male	5,741	2,389		
Marital status (n)	No	1739	729	14.562	<0.001
	Yes	10,249	4,411		
Regional categories (n)	East	4,177	1791	22.200	<0.001
	Midland	3,895	1,671		
	West	3,922	1,678		
Drinking status (n)	No	6,484	2,777	5.771	0.016
	Yes	5,483	2,349		
Education (n)	Elementary school or below	5,322	2,337	21.960	<0.001
	Secondary school	2,681	1,110		
	High school	2,578	1,074		
	University/College	1,410	619		
Hypertension (n)	No	7,555	3,229	7.095	0.008
	Yes	3,883	1,674		
Diabetes (n)	No	10,227	4,413	7.484	0.006
	Yes	1,144	460		

**Table 4:** Univariate and multivariate logistic regression analysis in individuals with glaucoma.

Variables	Univariate		Multivariate	
	*P*-value	OR (95% CI)	*P*-value	OR (95% CI)
Age	<0.001	1.010 [1.008, 1.012]	<0.001	1.682 [1.457, 1.942]
Gender	0.001	1.037 [1.016, 1.055]	0.021	0.692 [0.507, 0.945]
Regional categories	<0.001	1.049 [1.029, 1.068]	0.044	1.404 [1.111, 1.774]
Education	<0.001	1.044 [1.028, 1.068]	0.090	0.884 [0.766, 1.020]
Diabetes	0.008	1.028 [1.007, 1.050]	0.085	1.368 [0.958, 1.953]
NH_4_	0.035	1.004 [1.001, 1.007]	0.047	0.668 [0.449, 0.994]
NO_3_	0.042	1.012 [1.009, 1.015]	0.056	1.182 [0.996, 1.404]

### Efficiency analysis and internal validation of logistic regression model

**Figure 3:**
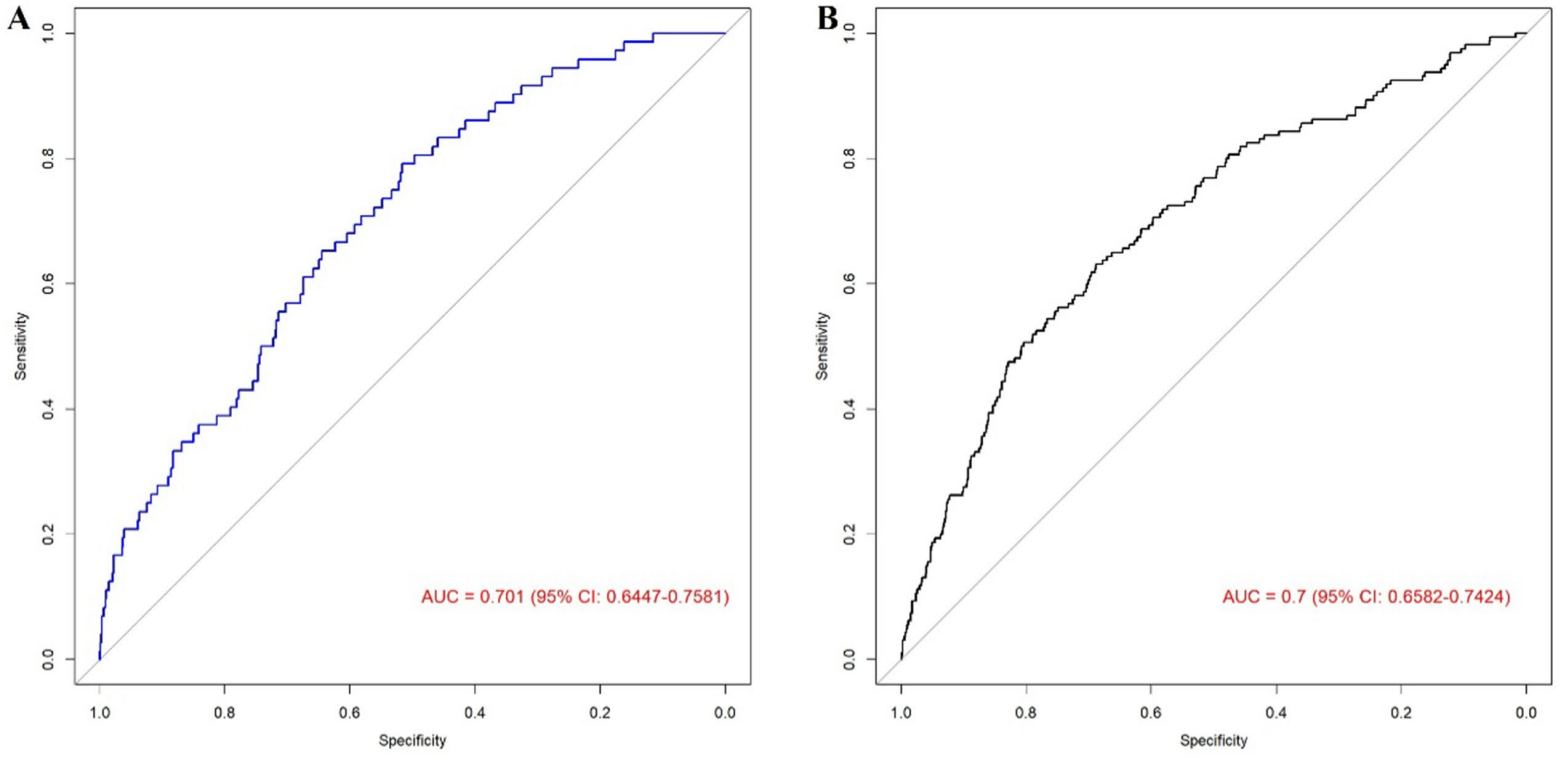
ROC curves: **(A)** Training, **(B)** Validation. **(A)** ROC curve of a logistic regression model in training cohort is used to test the ability of the model to distinguish between the glaucoma group and the non-glaucoma group. **(B)** ROC curve of a logistic regression model in validation cohort is used to test the ability of the model to distinguish between the glaucoma group and the non-glaucoma group.

**Figure 4:**
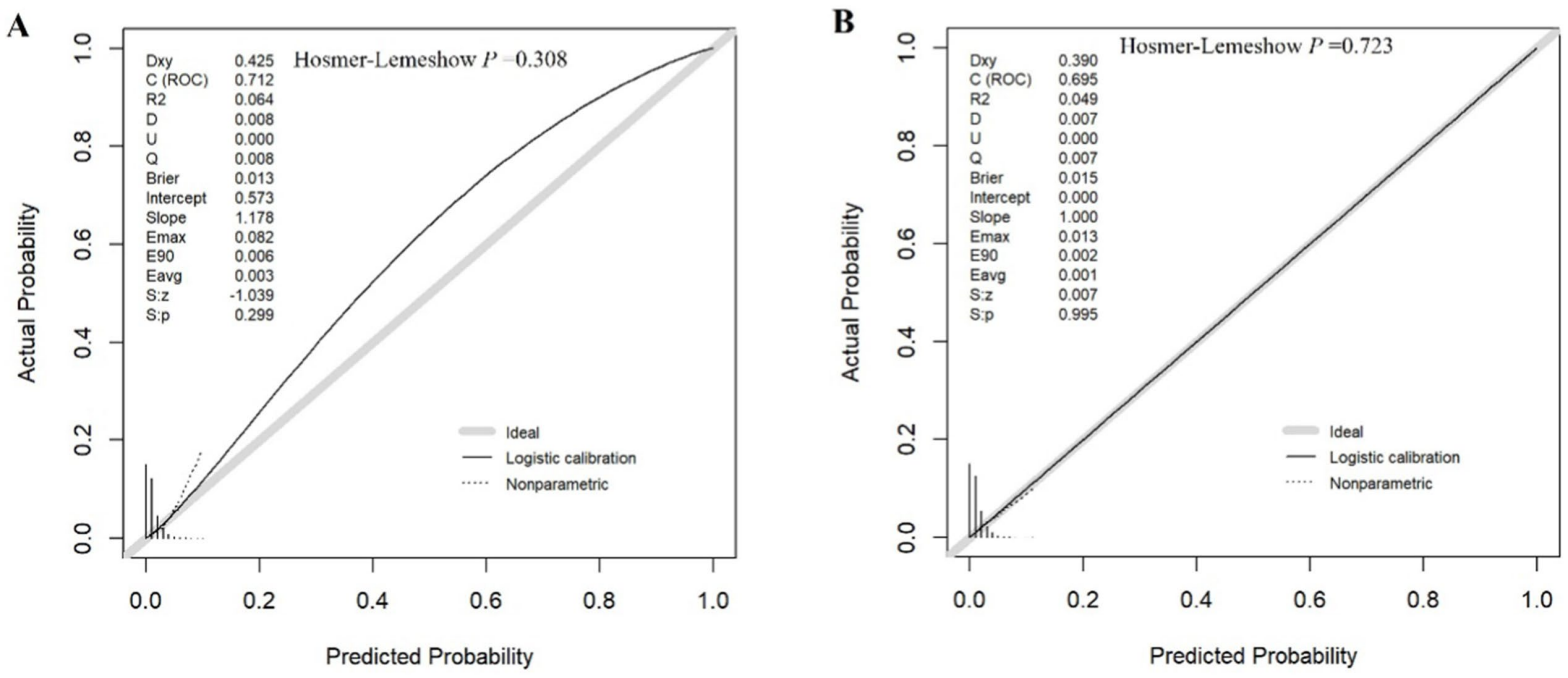
Calibration curves: **(A)** Training, **(B)** Validation. **(A)** The calibration curve of the training cohort of logistic regression model based on the bootstrap method, which is used to evaluate the probability accuracy of the model. **(B)** The calibration curve of the validation cohort of logistic regression based on the bootstrap method, which is used to evaluate the probability accuracy of the model.

As shown in Figure 3, in the training cohort, the AUC of the algorithm was 0.701 (95%CI: 0.6447–0.7581), the sensitivity was 0.650, and the specificity was 0.775. In the validation cohort, the AUC of the algorithm was 0.7 (95%CI, 0.6582–0.7424), the sensitivity was 0.658, and the specificity was 0.733. Subsequently, we perform internal validation by calibration curve. The internal validation of the model was carried out by the Bootstrap method. The original data were repeatedly sampled for 1,000 times. The AUC of the training cohort was 0.712, *χ*^2^ = 9.4249 in Hosmer-Lemeshow test (*p* = 0.308) and the Brier index was 0.013; the AUC of the validation cohort was 0.695, *χ*^2^ = 5.3161 in Hosmer–Lemeshow test (*p* = 0.723) and the Brier index was 0.015 (Figure 4), which showed that prediction model for glaucoma participants established by the present study had good performance among training cohort and validation cohort. These results indicate good discriminative and calibration performance of the model despite the inherent class imbalance in the dataset.

### Nomogram for estimating association between glaucoma and air pollutants as well as other factors

**Figure 5:**
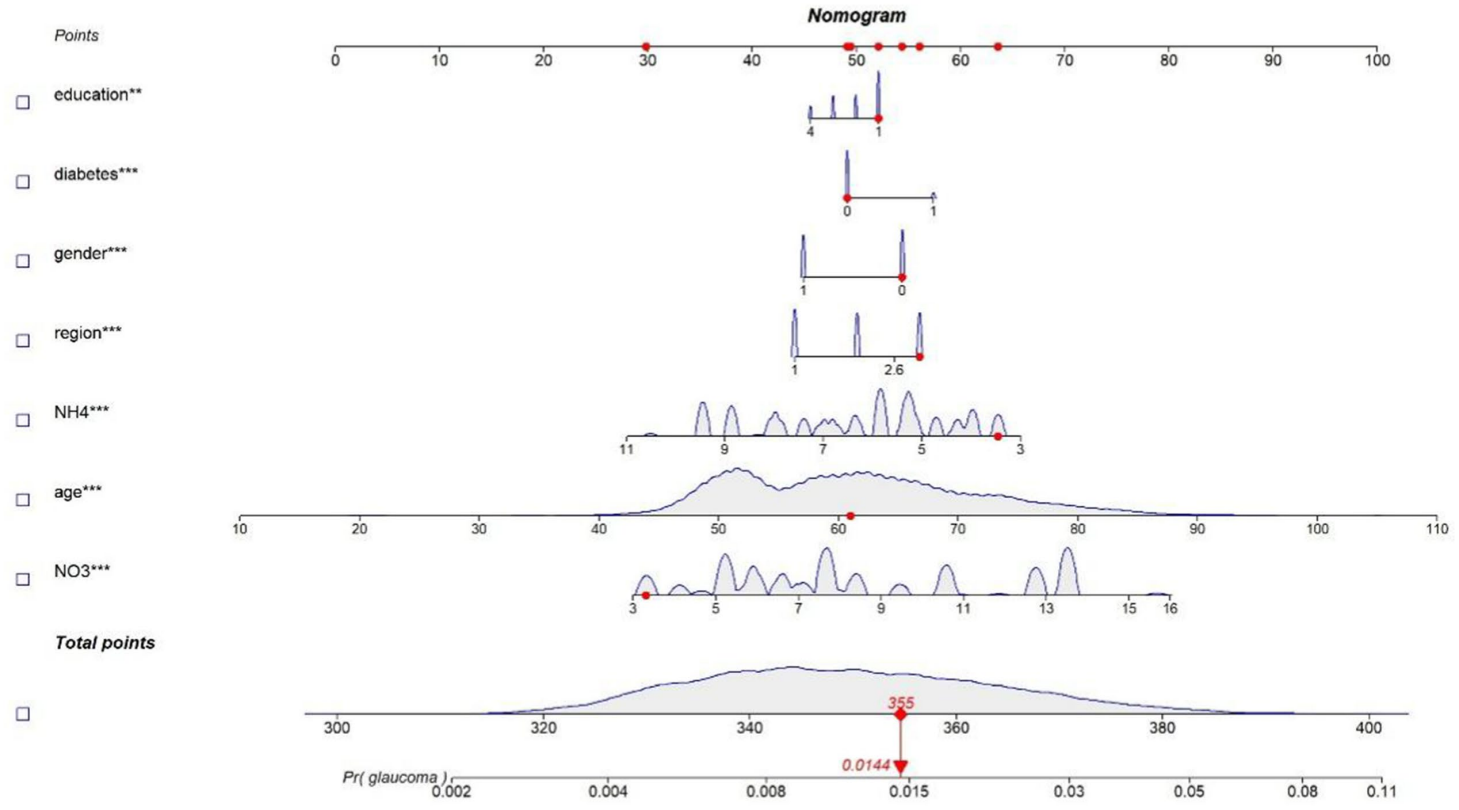
Nomogram of the predictive model. A novel nomogram to predict the prevalence of glaucoma. The nomogram provides a visual point system where each predictor variable is assigned a corresponding score on a common “Points” scale (top axis). The total score is calculated by summing the points for all variables, and this total is then mapped to the bottom scale to estimate the predicted probability of glaucoma. To calculate the probability of glaucoma, the points of seven variables determined on the scale were added to obtain the total points. Draw a vertical line from the total points scale to the last axis to obtain the corresponding probability of glaucoma.

**Figure 6:**
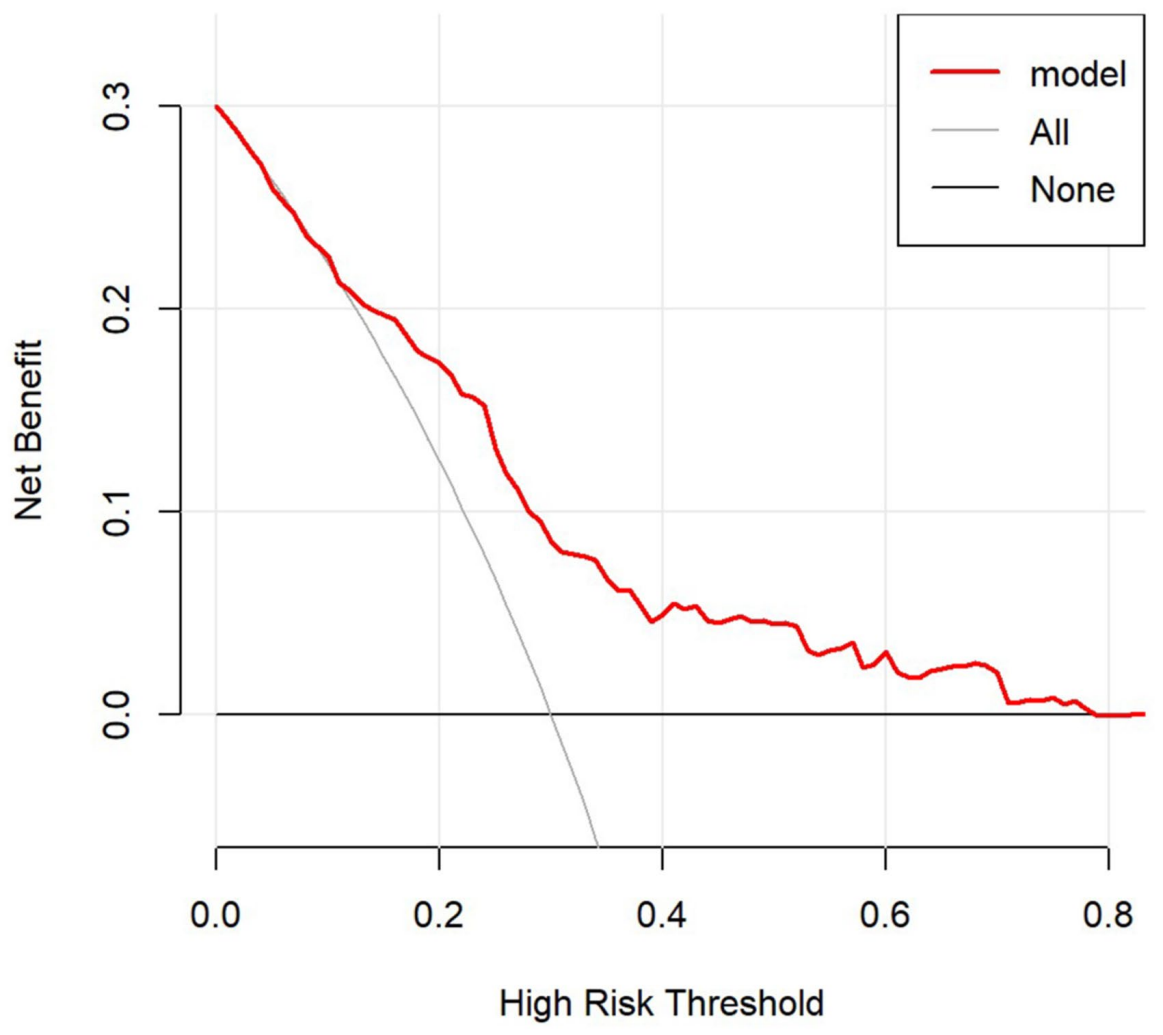
Decision curve analysis (DCA) for the nomogram. The DCA shows the clinical usefulness of the nomogram. The *Y*-axis represents net benefit. The bold solid black line is a nomogram predicting the risk of glaucoma. The solid gray line indicates that all patients occurred glaucoma, while the fine solid black line indicates that no patient occurred glaucoma. This DCA could provide a larger net benefit, with ranges of 0–80%.

The scores of each independent influencing factor were determined based on the CHARLS data, which was plotted in the nomogram of the association between glaucoma and air pollutants as well as other factors (Figure 5). The depicted DCA was used to determine whether decisions based on the predictive model had clinical applicability compared to the default strategy. The graphically DCA indicated the expected net benefit (red curve) per patient for predicting the risk of glaucoma. Within the threshold risk range of 0–80%, intervention decisions based on the predictive model are clearly beneficial (Figure 6).

## Discussion

Based on current knowledge, this study represents the first attempt to assess the association between air pollution, specifically particulate matter, and glaucoma among middle-aged and older adult populations. Air pollution exposure was estimated using satellite-based models, which provide regional estimates of pollution levels. Results indicate that environmental components of particulate matter, specifically NH₄ and NO₃, adversely impact glaucoma incidence. These substances are secondary inorganic aerosols that contribute to the overall composition of PM_2.5_. Furthermore, factors such as gender, age, educational level, diabetes, and regional categories may serve as predictive factors for glaucoma.

Epidemiological studies are increasingly focusing on the impact of air pollution exposure on various diseases. However, existing research predominantly centers on PM_2.5_ (13, 14), with fewer studies examining smaller particles such as NH_4_ and NO_3_. Sulfate (SO_4_^2−^), nitrate (NO_3_^−^), and ammonium (NH_4_^+^)—collectively termed SNA—account for 30 to 50% of PM_2.5_ concentration (15). NH_4_ and NO_3_ are PM_2.5_ components, formed as ammonia and nitric acid molecules transition from the gas phase to the liquid phase, ionizing to create ammonium nitrate (NH_4_NO_3_) (16). Approximately 70–80% of PM_2.5_ arises from secondary formation, with ammonium nitrate constituting a significant component, critical for particulate matter pollution control in several areas (17). Current research suggests a potential association between air pollution, particularly fine particulate matter (PM_2.5_), and glaucoma onset and progression (5, 18). Ammonium nitrate, as part of PM_2.5_, may be linked to glaucoma incidence. One possible mechanism is that ammonium nitrate combustion or atmospheric exposure releases nitrogen oxides, increasing oxidative stress and chronic inflammation—key factors in glaucoma pathology, especially regarding optic nerve damage (19, 20). Exposure to PM_2.5_ has been shown to elevate systemic oxidative stress and ocular inflammatory response (21), potentially exacerbating damage to the trabecular meshwork and retinal ganglion cells, thereby accelerating glaucoma progression. An *in vitro* study on human trabecular meshwork cells supports the biological plausibility of this association (18). Anatomically, particulate matter may induce the closure of a narrow anterior chamber angle, prompting angle-closure glaucoma (22).

In our model, regional classification functions as a crucial predictive factor among covariates. It is well-known that air pollutant density varies significantly by region, making it instructive to emphasize region-specific relationships between air pollutants and glaucoma. NH_4_ and NO_3_, as co-indicators, exhibit consistent regional distribution, with Tianjin, Shandong, and Jiangsu ranking as the most polluted cities (Supplementary Table S1). Air pollutant density positively correlates with industrialization levels, particularly in regions with heavy industries such as steel, chemicals, and petroleum processing (23, 24). In low-and middle-income countries industrialization often leads to significant environmental damage, which in turn drives social, economic, and lifestyle changes. These changes contribute to the rising prevalence of chronic diseases such as diabetes, myopia, and high blood pressure, particularly among the older adult population. Importantly, these chronic conditions are closely associated with an increased risk of glaucoma (25). Thus, industrialization indirectly elevates glaucoma risk by fostering environmental and lifestyle factors that predispose individuals to these chronic diseases. Geographically, Tianjin, Shandong, and Jiangsu are located in North and East China, situated on the eastern plains characterized by relatively flat terrain. This topography limits pollutant dispersion, particularly in winter when reduced cold air activity often leads to temperature inversions. During these inversions, temperature rises with altitude, causing pollutants to accumulate at lower altitudes and exacerbating air pollution. Furthermore, the humid air and coastal sea breezes in these areas can carry pollutants further inland, compounding pollution levels (26, 27). It is worth noting that Tianjin, the city with the highest concentration of NH_4_ and NO_3_, may also experience elevated glaucoma risk due to socioeconomic factors in addition to environmental pollution. Studies have shown that socioeconomic deprivation is an independent risk factor for advanced glaucoma, associated with poor education, limited access to healthcare, and perceived medical costs (28). As a city with a lower GDP index, Tianjin is particularly susceptible to these economic factors, which may interact synergistically with environmental pollution to further increase glaucoma risk.

Covariates such as age, gender, educational level, and diabetes play significant roles in glaucoma occurrence and progression, each contributing through distinct yet interconnected mechanisms. Age is a well-established risk factor, with glaucoma incidence rising exponentially after 40 (29). This is attributed to age-related degenerative changes in the optic nerve and reduced trabecular meshwork function, which impair aqueous humor outflow and elevate intraocular pressure (IOP) (30). Additionally, age-related vascular changes, such as reduced ocular blood flow, may exacerbate optic nerve damage (31). These findings align with global trends showing aging populations as a key driver of glaucoma prevalence. Gender-related effects vary by region and glaucoma subtype. For example, women may have a higher prevalence of primary open-angle glaucoma in some populations, potentially due to hormonal influences (32). In East Asia, women show higher rates of angle-closure glaucoma, likely due to shallower anterior chambers (33). These variations highlight the need for region-specific research to clarify gender’s role in glaucoma risk. Educational level indirectly influences glaucoma outcomes by shaping health behaviors and access to care. Higher education is associated with greater health literacy and participation in screening programs, enabling earlier detection (34). Conversely, lower educational attainment is linked to delayed diagnosis and advanced disease, underscoring the role of socioeconomic factors in glaucoma disparities. Diabetes impacts glaucoma risk through vascular and metabolic pathways. Reduced ocular blood flow and hyperglycemia-induced oxidative stress can impair optic nerve perfusion and elevate IOP (35). A meta-analysis showed that the overall relative risk of glaucoma in patients with and without diabetes was 1.48 (95% CI, 1.29–1.71), suggesting a bidirectional relationship where glaucoma may also hinder diabetes management (36). The interaction between environmental factors and regional characteristics is an important consideration. In highly industrialized regions like Tianjin, Shandong, and Jiangsu, environmental pollution and socioeconomic factors may synergistically increase glaucoma risk. Air pollutants such as PM_2.5_ and PM_10_ are linked to systemic inflammation and oxidative stress (37), potentially exacerbating glaucoma-related vascular and metabolic dysregulation (38). Regional disparities in healthcare access further compound these risks, particularly for vulnerable populations (39). In conclusion, these covariates interact through biological, behavioral, and environmental pathways to influence glaucoma risk. A deeper understanding of these mechanisms, particularly in understudied populations, is essential for developing targeted prevention and intervention strategies.

Although this study offers a novel perspective on the relationship between air pollutants and glaucoma, some limitations exist. First of all, although the investigator’s address is used to match the exposure indicators, the change of residence may lead to matching errors. Second, although our study controls for several important confounders, it fails to account for potential confounders such as household clean fuel use. Finally, this study was cross-sectional in nature, using exposure and health outcome data from a single year (2015). As a result, we could not assess temporal relationships or causal inferences. Future longitudinal studies with repeated measures of both air pollution and glaucoma diagnoses are warranted to confirm our findings and further explore the temporal dynamics of the association.

## Conclusion

In our study, we examined the role of environmental pollutants, specifically NH_4_ and NO_3_, which are key components of secondary inorganic aerosols contributing to PM_2.5_. These compounds form through atmospheric reactions involving NH₃ and NO_4_, particularly in areas with high industrial and agricultural activities. Our findings reveal a significant association between elevated levels of NH₄ and NO₃ and increased glaucoma incidence among middle-aged and older adult populations in China. This association is most pronounced in industrialized regions like Tianjin, Shandong, and Jiangsu. By focusing on NH₄ and NO₃ as components of PM_2.5_, our study underscores the need to address secondary inorganic aerosols in air pollution control strategies. Reducing emissions of their precursors, such as NH₃ and NO_4_, could significantly lower PM_2.5_ levels and, in turn, reduce associated health risks, including glaucoma. Future research should investigate the specific mechanisms by which these components contribute to glaucoma pathogenesis, as well as explore potential synergistic effects with other air pollutants and environmental factors.

### Patient and public involvement

It was not appropriate or possible to involve patients or the public in the design, or conduct, or reporting, or dissemination plans of our research.

## Data Availability

Publicly available datasets were analyzed in this study. This data can be found: https://charls.pku.edu.cn/.

## Footnotes

^1^ http://charls.pku.edu.cn

^2^ https://weijing-rs.github.io/product.html
