# Supplementary material for: Exploring association between ambient air pollution and glaucoma in China: a nationwide analysis with predictive modeling based on the China Health and Retirement Longitudinal Study
Source: Front Public Health. 2025 Apr 25;13:1541803. doi: 10.3389/fpubh.2025.1541803 (PMC12061970; doi:10.3389/fpubh.2025.1541803)
Supplement: Supplementary file 1 [file Table_1.docx]

**Table S1**

PM_1_ concentration in China

PM_2.5_ concentration in China

| Province | Mean concentration | Max concentration | Min concentration | Sd concentration |
| --- | --- | --- | --- | --- |
| Anhui | 29.1765165 | 44.0000007 | 16.1000002 | 5.328265 |
| Beijing | 35.2822478 | 55.0000008 | 21.9000003 | 7.82643592 |
| Chongqing | 20.7437179 | 40.9000006 | 12.8000002 | 4.0616532 |
| Fujian | 17.3870273 | 28.8000004 | 10.7000002 | 1.59398678 |
| Gansu | 21.3642409 | 34.2000005 | 10.7000002 | 2.73460873 |
| Guangdong | 19.8842616 | 29.0000004 | 13.6000002 | 1.53130519 |
| Guangxi | 20.2266913 | 30.8000005 | 14.9000002 | 1.9238447 |
| Guizhou | 16.8197219 | 27.7000004 | 9.40000014 | 1.28935357 |
| Hebei | 33.4906198 | 63.4000009 | 15.7000002 | 11.8140385 |
| Heilongjiang | 20.7260845 | 44.0000007 | 11.3000002 | 5.25028641 |
| Henan | 39.10767 | 60.7000009 | 22.2000003 | 6.64198413 |
| Hubei | 27.2420658 | 49.1000007 | 9.60000014 | 7.15348503 |
| Hunan | 23.3442556 | 34.9000005 | 14.7000002 | 3.95554912 |
| Inner Mongol | 16.6656403 | 31.8000005 | 8.10000012 | 4.17732582 |
| Jiangsu | 31.7966463 | 46.0000007 | 19.5000003 | 3.68227439 |
| Jiangxi | 22.1390539 | 35.8000005 | 13.8000002 | 3.33990853 |
| Jilin | 25.7061917 | 37.1000006 | 16.9000003 | 4.39310177 |
| Liaoning | 28.8508042 | 48.2000007 | 20.3000003 | 3.55324017 |
| Qinghai | 18.0640012 | 28.0000004 | 6.4000001 | 3.01810626 |
| Shaanxi | 23.9500335 | 46.2000007 | 16.1000002 | 4.41826684 |
| Shandong | 38.3243559 | 59.1000009 | 18.9000003 | 6.86877077 |
| Shanghai | 27.3078315 | 38.5000006 | 17.3000003 | 3.07629499 |
| Shanxi | 28.7843974 | 54.0000008 | 19.9000003 | 5.06486493 |
| Sichuan | 16.5246517 | 44.1000007 | 4.50000007 | 7.75438415 |
| Tianjin | 41.9502976 | 54.7000008 | 31.2000005 | 2.08770623 |
| Xinjiang | 21.0521981 | 36.7000005 | 6.10000009 | 4.81100539 |
| Yunnan | 15.3340585 | 25.7000004 | 4.00000006 | 3.68917987 |
| Zhejiang | 23.9708098 | 41.2000006 | 14.6000002 | 5.02885931 |

| Province | Mean concentration | Max concentration | Min concentration | Sd concentration |
| --- | --- | --- | --- | --- |
| Anhui | 52.2756069 | 74.0000011 | 26.9000004 | 9.83440455 |
| Beijing | 64.1959113 | 90.7000014 | 40.4000006 | 13.5173434 |
| Chongqing | 42.9743686 | 64.100001 | 29.1000004 | 7.09071423 |
| Fujian | 28.2363302 | 45.8000007 | 18.2000003 | 2.08060876 |
| Gansu | 40.2172742 | 60.1000009 | 24.5000004 | 4.68709863 |
| Guangdong | 32.9023441 | 46.0000007 | 22.8000003 | 2.64008137 |
| Guangxi | 35.6969663 | 54.8000008 | 27.8000004 | 3.02103527 |
| Guizhou | 32.91805 | 52.3000008 | 17.3000003 | 3.10837809 |
| Hebei | 61.5015672 | 114.000002 | 29.4000004 | 21.8509196 |
| Heilongjiang | 35.7472496 | 77.2000012 | 21.3000003 | 6.53850863 |
| Henan | 68.3770799 | 102.400002 | 39.6000006 | 11.387217 |
| Hubei | 50.8359053 | 79.9000012 | 29.6000004 | 11.4369685 |
| Hunan | 43.9695982 | 64.600001 | 27.2000004 | 6.99246356 |
| Inner Mongol | 34.6556756 | 60.1000009 | 16.7000002 | 6.39167771 |
| Jiangsu | 57.3603687 | 78.7000012 | 34.1000005 | 6.57999635 |
| Jiangxi | 37.0130786 | 66.000001 | 23.9000004 | 5.09706926 |
| Jilin | 44.3297408 | 68.600001 | 30.8000005 | 7.23010276 |
| Liaoning | 49.5676868 | 81.5000012 | 37.0000006 | 6.05437314 |
| Qinghai | 39.8143043 | 57.3000009 | 18.9000003 | 4.98749404 |
| Shaanxi | 42.3315785 | 77.6000012 | 29.4000004 | 7.18764329 |
| Shandong | 70.4949469 | 105.500002 | 33.7000005 | 12.7173606 |
| Shanghai | 49.1310513 | 68.300001 | 39.0000006 | 3.95440348 |
| Shanxi | 49.6596001 | 87.5000013 | 37.1000006 | 7.64702164 |
| Sichuan | 32.8397401 | 79.6000012 | 15.5000002 | 10.6110362 |
| Tianjin | 76.0472482 | 91.2000014 | 57.4000009 | 3.72959385 |
| Xinjiang | 55.3167074 | 129.500002 | 10.4000002 | 21.7174617 |
| Yunnan | 26.0766358 | 50.0000007 | 14.0000002 | 3.74546804 |
| Zhejiang | 38.1274348 | 65.800001 | 24.8000004 | 7.83034828 |

PM_10_ concentration in China

| Province | Mean concentration | Max concentration | Min concentration | Sd concentration |
| --- | --- | --- | --- | --- |
| Anhui | 81.9765295 | 122.100002 | 46.7000007 | 15.4387625 |
| Beijing | 106.325286 | 144.100002 | 76.2000011 | 16.9201032 |
| Chongqing | 67.7482802 | 105.100002 | 46.7000007 | 10.3536576 |
| Fujian | 45.8693069 | 64.900001 | 30.4000005 | 3.02276041 |
| Gansu | 102.125672 | 174.800003 | 43.7000007 | 25.3930268 |
| Guangdong | 50.8944978 | 67.900001 | 38.4000006 | 3.5655053 |
| Guangxi | 54.3417466 | 84.5000013 | 45.3000007 | 3.63609821 |
| Guizhou | 53.1099229 | 86.4000013 | 38.3000006 | 5.15029567 |
| Hebei | 110.095393 | 188.600003 | 62.8000009 | 31.0492164 |
| Heilongjiang | 58.3094491 | 130.000002 | 37.0000006 | 8.71232191 |
| Henan | 114.052945 | 187.700003 | 66.400001 | 19.3310853 |
| Hubei | 80.2037648 | 124.600002 | 49.1000007 | 17.2141477 |
| Hunan | 68.5923702 | 101.400002 | 44.3000007 | 10.9257084 |
| Inner Mongol | 81.9514578 | 148.200002 | 44.3000007 | 25.1990894 |
| Jiangsu | 93.4061181 | 134.500002 | 61.4000009 | 12.0276093 |
| Jiangxi | 57.6414518 | 96.8000014 | 43.4000006 | 8.12150255 |
| Jilin | 74.1722842 | 120.500002 | 51.2000008 | 10.4566067 |
| Liaoning | 84.6400032 | 136.100002 | 66.400001 | 9.25797449 |
| Qinghai | 94.2008856 | 166.400002 | 47.4000007 | 19.9905759 |
| Shaanxi | 82.8277249 | 143.800002 | 48.0000007 | 17.5741945 |
| Shandong | 120.450333 | 188.600003 | 58.7000009 | 20.4785692 |
| Shanghai | 75.5566237 | 100.900002 | 61.5000009 | 5.6839507 |
| Shanxi | 94.8803832 | 135.900002 | 70.200001 | 9.48558617 |
| Sichuan | 60.5076153 | 121.800002 | 30.3000005 | 14.0260563 |
| Tianjin | 128.279255 | 145.400002 | 98.2000015 | 5.35176346 |
| Xinjiang | 165.2797 | 386.500006 | 28.1000004 | 93.0154987 |
| Yunnan | 46.4199088 | 79.3000012 | 25.4000004 | 5.13370594 |
| Zhejiang | 60.021718 | 96.8000014 | 41.3000006 | 11.2896989 |

O_3_ concentration in China

| Province | Mean concentration | Max concentration | Min concentration | Sd concentration |
| --- | --- | --- | --- | --- |
| Anhui | 73.1040348 | 109.500002 | 29.4000004 | 7.91881993 |
| Beijing | 92.1085833 | 103.200002 | 85.6000013 | 2.22361105 |
| Chongqing | 75.309101 | 96.2000014 | 47.7000007 | 3.32856605 |
| Fujian | 74.1775917 | 110.500002 | 47.6000007 | 4.52560797 |
| Gansu | 90.4429461 | 115.300002 | 67.400001 | 6.65965085 |
| Guangdong | 78.8928339 | 117.500002 | 39.5000006 | 5.45910436 |
| Guangxi | 75.9685521 | 97.0000014 | 53.1000008 | 3.52937414 |
| Guizhou | 74.8016367 | 88.6000013 | 44.6000007 | 2.88524066 |
| Hebei | 89.8399546 | 109.800002 | 54.7000008 | 4.09219754 |
| Heilongjiang | 76.4963058 | 100.400001 | 47.7000007 | 7.2035032 |
| Henan | 85.4655401 | 112.500002 | 55.9000008 | 3.80065843 |
| Hubei | 82.4968971 | 114.000002 | 44.7000007 | 6.28485046 |
| Hunan | 75.9695785 | 110.900002 | 39.9000006 | 4.61558368 |
| Inner Mongol | 84.5161201 | 113.700002 | 43.9000007 | 11.2141205 |
| Jiangsu | 95.3716269 | 124.700002 | 36.8000005 | 7.16860286 |
| Jiangxi | 71.5907105 | 98.6000015 | 43.7000007 | 3.69466757 |
| Jilin | 87.7449641 | 110.700002 | 68.500001 | 4.91054267 |
| Liaoning | 86.9482678 | 120.200002 | 28.1000004 | 7.51250468 |
| Qinghai | 80.2877652 | 117.900002 | 56.2000008 | 14.1840679 |
| Shaanxi | 86.2166889 | 105.200002 | 52.5000008 | 6.54180246 |
| Shandong | 96.028222 | 127.800002 | 60.0000009 | 4.94755426 |
| Shanghai | 99.0969453 | 111.200002 | 87.2000013 | 3.18686029 |
| Shanxi | 88.5923111 | 122.900002 | 43.8000007 | 4.05270001 |
| Sichuan | 75.5248818 | 115.700002 | 35.2000005 | 6.34136118 |
| Tianjin | 87.582447 | 101.100002 | 66.300001 | 4.1749799 |
| Xinjiang | 83.8269299 | 107.400002 | 36.9000005 | 9.87517176 |
| Yunnan | 80.6689441 | 120.300002 | 42.3000006 | 5.49876375 |
| Zhejiang | 83.2975945 | 114.100002 | 62.7000009 | 8.21395047 |

NH_4_ concentration in China

| Province | Mean concentration | Max concentration | Min concentration | Sd concentration |
| --- | --- | --- | --- | --- |
| Anhui | 7.39807541 | 9.46999979 | 3.99999991 | 1.21431417 |
| Beijing | 8.3211421 | 13.5999997 | 5.10999989 | 1.31326311 |
| Chongqing | 6.44504671 | 8.7699998 | 4.00999991 | 0.69410228 |
| Fujian | 4.23646616 | 6.99999984 | 3.15999993 | 0.25908688 |
| Gansu | 3.99081213 | 6.63999985 | 0 | 0.83562999 |
| Guangdong | 4.71148148 | 6.84999985 | 2.89999994 | 0.40252462 |
| Guangxi | 5.37512383 | 6.63999985 | 3.65999992 | 0.35734522 |
| Guizhou | 5.08838762 | 7.30999984 | 2.63999994 | 0.85275684 |
| Hebei | 7.88364975 | 16.2099996 | 3.77999992 | 2.54496103 |
| Heilongjiang | 4.34201635 | 5.84999987 | 0 | 1.2689594 |
| Henan | 8.86648869 | 14.6699997 | 5.56999988 | 0.96823513 |
| Hubei | 7.06832327 | 10.8299998 | 4.15999991 | 0.85561445 |
| Hunan | 6.33198735 | 8.00999982 | 4.4199999 | 0.52123777 |
| Inner Mongol | 3.95981511 | 7.64999983 | 0 | 0.67726736 |
| Jiangsu | 8.05277427 | 10.4899998 | 5.12999989 | 0.87652416 |
| Jiangxi | 5.2892445 | 7.72999983 | 3.89999991 | 0.58019114 |
| Jilin | 5.30519949 | 7.00999984 | 0 | 1.02574544 |
| Liaoning | 6.73128156 | 10.6999998 | 4.84999989 | 0.74443082 |
| Shaanxi | 5.83174319 | 10.3199998 | 3.57999992 | 1.25411432 |
| Shandong | 9.44818117 | 15.2199997 | 5.00999989 | 1.3245839 |
| Shanghai | 6.24251517 | 8.8399998 | 4.4699999 | 0.47227356 |
| Shanxi | 6.89629047 | 12.6099997 | 4.02999991 | 1.70711756 |
| Sichuan | 5.84646543 | 9.50999979 | 0 | 1.63453555 |
| Tianjin | 10.5104475 | 12.3799997 | 8.01999982 | 0.50491517 |
| Yunnan | 3.45429787 | 7.03999984 | 0 | 1.27828465 |
| Zhejiang | 5.16981407 | 8.26999982 | 3.58999992 | 0.80546154 |

NO_3_ concentration in China

| Province | Mean concentration | Max concentration | Min concentration | Sd concentration |
| --- | --- | --- | --- | --- |
| Anhui | 10.6099919 | 18.4499996 | 4.08999991 | 2.94425426 |
| Beijing | 11.8471312 | 18.9199996 | 6.67999985 | 2.67877824 |
| Chongqing | 7.9407764 | 12.4399997 | 4.82999989 | 1.7468215 |
| Fujian | 4.12280356 | 11.5199997 | 2.73999994 | 0.67969429 |
| Gansu | 5.76276099 | 8.32999981 | 0 | 0.97933142 |
| Guangdong | 5.20999566 | 10.2299998 | 3.09999993 | 0.65667413 |
| Guangxi | 5.28230245 | 8.64999981 | 3.02999993 | 0.63946375 |
| Guizhou | 4.64925168 | 9.63999978 | 2.30999995 | 1.07379595 |
| Hebei | 10.5330405 | 22.2199995 | 4.78999989 | 3.9551621 |
| Heilongjiang | 5.94156797 | 8.21999982 | 0 | 1.74263513 |
| Henan | 12.7436533 | 18.1199996 | 6.65999985 | 1.83425376 |
| Hubei | 9.45364778 | 16.2199996 | 4.83999989 | 2.36946905 |
| Hunan | 7.64261139 | 12.0099997 | 4.03999991 | 1.29438421 |
| Inner Mongol | 5.20696631 | 9.81999978 | 0 | 0.85159527 |
| Jiangsu | 13.4721158 | 18.1199996 | 8.8899998 | 1.10770808 |
| Jiangxi | 5.98082574 | 12.6299997 | 2.73999994 | 1.32645814 |
| Jilin | 6.7342529 | 10.0399998 | 0 | 1.27735394 |
| Liaoning | 8.39900949 | 15.2599997 | 6.03999987 | 1.20560305 |
| Shaanxi | 7.11530045 | 15.2299997 | 4.99999989 | 1.65934964 |
| Shandong | 13.5302239 | 19.7399996 | 6.83999985 | 2.00111703 |
| Shanghai | 10.8476343 | 16.1499996 | 8.39999981 | 1.09191589 |
| Shanxi | 8.39492273 | 15.5699997 | 5.59999987 | 1.77966006 |
| Sichuan | 7.67071163 | 14.9299997 | 0 | 2.25953844 |
| Tianjin | 15.6671138 | 18.4299996 | 10.8699998 | 0.94865496 |
| Yunnan | 3.32262206 | 9.23999979 | 0 | 1.23870076 |
| Zhejiang | 6.5473718 | 15.4599997 | 3.41999992 | 2.58303646 |

SO_4_ concentration in China

| Province | Mean concentration | Max concentration | Min concentration | Sd concentration |
| --- | --- | --- | --- | --- |
| Anhui | 10.5772998 | 14.0499997 | 6.47999986 | 1.28020074 |
| Beijing | 11.6463518 | 18.5399996 | 7.85999982 | 1.98480976 |
| Chongqing | 10.9596778 | 13.3799997 | 8.61999981 | 0.79872703 |
| Fujian | 7.69984716 | 10.5399998 | 5.68999987 | 0.38972789 |
| Gansu | 8.31538349 | 11.4999997 | 0 | 1.41006867 |
| Guangdong | 8.80166114 | 10.6599998 | 6.29999986 | 0.57986979 |
| Guangxi | 9.3869314 | 11.6999997 | 6.72999985 | 0.4990344 |
| Guizhou | 9.29526442 | 12.0499997 | 5.18999988 | 0.83222312 |
| Hebei | 11.9363348 | 24.4399995 | 6.11999986 | 3.91596666 |
| Heilongjiang | 5.95013938 | 8.31999981 | 0 | 1.76110275 |
| Henan | 14.5991058 | 21.0699995 | 10.4399998 | 1.61664518 |
| Hubei | 12.1968099 | 18.3099996 | 8.9099998 | 1.47234849 |
| Hunan | 11.3054011 | 14.7199997 | 8.29999981 | 1.12363306 |
| Inner Mongol | 6.92932413 | 11.6899997 | 0 | 1.16313581 |
| Jiangsu | 11.1257931 | 14.8899997 | 7.32999984 | 1.0283141 |
| Jiangxi | 9.17987667 | 14.1299997 | 6.59999985 | 0.83063097 |
| Jilin | 6.96836153 | 9.0099998 | 0 | 1.32171931 |
| Liaoning | 8.18415551 | 14.5999997 | 6.33999986 | 0.79959409 |
| Shaanxi | 10.1867288 | 15.8799996 | 7.55999983 | 1.35427674 |
| Shandong | 13.363593 | 20.6599995 | 6.90999985 | 2.30035061 |
| Shanghai | 9.82329014 | 12.9399997 | 8.42999981 | 0.63462406 |
| Shanxi | 12.1580171 | 18.8499996 | 8.42999981 | 2.10157679 |
| Sichuan | 9.8683974 | 13.8199997 | 0 | 2.24747312 |
| Tianjin | 14.1430514 | 17.0599996 | 10.5599998 | 0.79326721 |
| Yunnan | 7.23035764 | 11.1699998 | 0 | 2.20900961 |
| Zhejiang | 8.58032416 | 11.8399997 | 6.59999985 | 0.97660969 |

Cl concentration in China

| Province | Mean concentration | Max concentration | Min concentration | Sd concentration |
| --- | --- | --- | --- | --- |
| Anhui | 1.68107928 | 3.02999993 | 0.79999998 | 0.38696773 |
| Beijing | 2.63184697 | 4.77999989 | 1.79999996 | 0.50022516 |
| Chongqing | 1.27877242 | 2.77999994 | 0.94999998 | 0.18955967 |
| Fujian | 0.87163574 | 2.76999994 | 0.61999999 | 0.11256796 |
| Gansu | 1.80436231 | 3.78999992 | 0 | 0.38219159 |
| Guangdong | 1.01833766 | 3.52999992 | 0.68999998 | 0.21067441 |
| Guangxi | 0.92003613 | 2.26999995 | 0.44999999 | 0.08776491 |
| Guizhou | 0.91658741 | 1.60999996 | 0.64999999 | 0.11390529 |
| Hebei | 2.98606798 | 8.08999982 | 1.44999997 | 0.91852185 |
| Heilongjiang | 1.93039985 | 2.93999993 | 0 | 0.5694116 |
| Henan | 2.69690226 | 6.88999985 | 1.19999997 | 0.68286178 |
| Hubei | 1.4969811 | 3.65999992 | 0.99999998 | 0.28136782 |
| Hunan | 1.17274571 | 2.75999994 | 0.87999998 | 0.12274289 |
| Inner Mongol | 1.98516215 | 3.90999991 | 0 | 0.25257448 |
| Jiangsu | 2.10198832 | 4.18999991 | 1.32999997 | 0.29271611 |
| Jiangxi | 1.09068919 | 5.78999987 | 0.74999998 | 0.15832201 |
| Jilin | 2.16781202 | 3.81999991 | 0 | 0.43910381 |
| Liaoning | 2.48117615 | 5.97999987 | 1.79999996 | 0.35064431 |
| Shaanxi | 1.86108968 | 4.11999991 | 1.08999998 | 0.32311111 |
| Shandong | 3.32324337 | 9.1199998 | 1.77999996 | 0.61368575 |
| Shanghai | 1.76324682 | 4.07999991 | 1.22999997 | 0.2658082 |
| Shanxi | 2.54626362 | 4.6599999 | 1.85999996 | 0.32830787 |
| Sichuan | 1.66218718 | 4.04999991 | 0 | 0.45126504 |
| Tianjin | 3.85494627 | 6.72999985 | 2.58999994 | 0.34325323 |
| Yunnan | 0.94420618 | 2.31999995 | 0 | 0.30792028 |
| Zhejiang | 1.27520611 | 3.27999993 | 0.77999998 | 0.30560101 |

**Table S2** Association between air pollution and the prevalence of glaucoma

| Variable | OR (95% CI) | P for interaction |
| --- | --- | --- |
| O_3_ |  |  |
| model1 | 1.000[1.000-1.000] | 0.04 |
| model2 | 1.000[1.000-1.001] | 0.379 |
| model3 | 1.000[1.000-1.002] | 0.248 |
| PM_2.5_ |  |  |
| model1 | 1.000[0.999-1.000] | 0.206 |
| model2 | 1.208[1.134-1.288] | 0.005 |
| model3 | 1.221[1.145-1.301] | 0.003 |
| PM_10_ |  |  |
| model1 | 1.005[0.997-1.010] | 0.11 |
| model2 | 1.123[1.050-1.201] | 0.005 |
| model3 | 1.117[1.042-1.195] | 0.009 |
| SO_4_ |  |  |
| model1 | 1.000[0.999-1.000] | 0.746 |
| model2 | 1.000[0.999-1.001] | 0.793 |
| model3 | 1.000[0.999-1.001] | 0.684 |
| PM_1_ |  |  |
| model1 | 1.001[0.998-1.002] | 0.304 |
| model2 | 1.299[1.208-1.397] | 0.004 |
| model3 | 1.303[1.211-1.403] | 0.002 |
| NH_4_ |  |  |
| model1 | 1.004[0.999-1.006] | 0.348 |
| model2 | 1.095[1.023-1.155] | 0.035 |
| model3 | 1.116[1.046-1.210] | 0.025 |
| Cl |  |  |
| model1 | 1.001[0.998-1.003] | 0.325 |
| model2 | 1.105[1.045-1.169] | 0.015 |
| model3 | 1.097[1.036-1.161] | 0.018 |
| NO_3_ |  |  |
| model1 | 1.001[0.997-1.003] | 0.171 |
| model2 | 1.168[1.085-1.247] | 0.009 |
| model3 | 1.172[1.099-1.250] | 0.009 |
